# Severe Perinatal Hypoxic-Ischemic Brain Injury Induces Long-Term Sensorimotor Deficits, Anxiety-Like Behaviors and Cognitive Impairment in a Sex-, Age- and Task-Selective Manner in C57BL/6 Mice but Can Be Modulated by Neonatal Handling

**DOI:** 10.3389/fnbeh.2019.00007

**Published:** 2019-02-13

**Authors:** Aida Muntsant, Kalpana Shrivastava, Mireia Recasens, Lydia Giménez-Llort

**Affiliations:** ^1^Department of Psychiatry and Forensic Medicine, School of Medicine, Universitat Autònoma de Barcelona, Barcelona, Spain; ^2^Institut de Neurociències, Universitat Autònoma de Barcelona, Barcelona, Spain; ^3^Department of Cell Biology, Physiology & Immunology, Universitat Autònoma de Barcelona, Barcelona, Spain

**Keywords:** neonatal hypoxic ischemic injury, neonatal handling, sensory stimulation, animal model, sex, gender medicine, behavior, cognition

## Abstract

Perinatal brain injury (PBI) leads to neurological disabilities throughout life, from motor deficits, cognitive limitations to severe cerebral palsy. Yet, perinatal brain damage has limited therapeutic outcomes. Besides, the immature brain of premature children is at increased risk of hypoxic/ischemic (HI) injury, with males being more susceptible to it and less responsive to protective/therapeutical interventions. Here, we model in male and female C57BL/6 mice, the impact of neonatal HI and the protective effects of neonatal handling (NH), an early life tactile and proprioceptive sensory stimulation. From postnatal day 1 (PND1, modeling pre-term) to PND21 randomized litters received either NH or left undisturbed. HI brain damage occurred by permanent left carotid occlusion followed by hypoxia at PND7 (modeling full-term) in half of the animals. The behavioral and functional screening of the pups at weaning (PND23) and their long-term outcomes (adulthood, PND70) were evaluated in a longitudinal study, as follows: somatic development (weight), sensorimotor functions (reflexes, rods and hanger tests), exploration [activity (ACT) and open-field (OF) test], emotional and anxiety-like behaviors [corner, open-field and dark-light box (DLB) tests], learning and memory [T-maze (TM) and Morris Water-Maze (MWM)]. HI induced similar brain damage in both sexes but affected motor development, sensorimotor functions, induced hyperactivity at weaning, and anxiety-like behaviors and cognitive deficits at adulthood, in a sex- and age-dependent manner. Thus, during ontogeny, HI affected equilibrium especially in females and prehensility in males, but only reflexes at adulthood. Hyperactivity of HI males was normalized at adulthood. HI increased neophobia and other anxiety-like behaviors in males but emotionality in females. Both sexes showed worse short/long-term learning, but memory was more affected in males. Striking neuroprotective effects of NH were found, with significantly lower injury scores, mostly in HI males. At the functional level, NH reversed the impaired reflex responses and improved memory performances in hippocampal-dependent spatial-learning tasks, especially in males. Finally, neuropathological correlates referred to atrophy, neuronal densities and cellularity in the affected areas [hippocampal-CA, caudate/putamen, thalamus, neocortex and corpus callosum (CC)] point out distinct neuronal substrates underlying the sex- and age- functional impacts of these risk/protection interventions on sensorimotor, behavioral and cognitive outcomes from ontogeny to adulthood.

## Introduction

Perinatal brain injury (PBI) due to hypoxia-ischemia (HI) is such a devastating early insult that it is considered to be a major contributor to perinatal morbidity and mortality. The prevalence of neonatal HI encephalopathy (HIE) due to an oxygen and glucose deprivation during birth is 1.5–3 and up to 6 per 1,000 livebirths in developed and developing countries, respectively (Kurinczuk et al., 2010). The perinatal brain is highly susceptible to damage due to the prevailing development processes. The most vulnerable regions to injury are the ones with greatest metabolic demands (sensorimotor cortex and basal ganglia, thalamus, cerebellum and brainstem; Thorngren-Jerneck et al., 2001). Consequently, PBI can lead to long-term neurologic disability in both children and adults, including cognitive limitations, learning difficulties, attention or motor deficits (van Handel et al., 2007) and even cerebral palsy, and seizures (Platt et al., 2007). Despite the improvements in neonatal care, brain damage in term newborn infants still remains a clinical problem, with research constrained by obvious ethical limitations. Most importantly, the immature brain of premature children is at increased risk of hypoxic ischemic injury (Vannucci and Hagberg, 2004) with males born prematurely being reported as most susceptible to it and with worse developmental and adult outcomes (Elsmén et al., 2004; Peacock et al., 2012; Månsson et al., 2015).

It is considered that the experimental model of HI-induced neonatal injury initially described by Vannucci and Vannucci (1997) for the rat (Rice et al., 1981), and also adapted to the mouse in several laboratories (Sheldon et al., 1998; Hagberg et al., 2002; Northington, 2006) is a useful translational technique to better understand the effects of HI injury in human brain. Moreover, this technique at postnatal day (PND) 7–10 is equivalent to a term human infant (Semple et al., 2013; Mallard and Vexler, 2015). Although many experimental studies, mostly in rats, report morphological, biophysical and biochemical changes following HI brain insult (Towfighi et al., 1991; Huang and Castillo, 2008; Shrivastava et al., 2012), there is a scarcity of data to understand the consequential behavioral changes. However, some reports do suggest that injured animals exhibited sensorimotor deficits (i.e., Jansen and Low, 1996; Bona et al., 1997) and suffered certain learning disabilities (i.e., Young et al., 1986; Balduini et al., 2000; Ikeda et al., 2001; McAuliffe et al., 2006). Sex differences regarding the final outcome after an adult stroke or after neonatal HI had been documented (Bona et al., 1998; Hagberg et al., 2004; Hurn et al., 2005; Smith et al., 2014; Netto et al., 2017). These differences are justified by the presence of sex-specific hormones that may influence the consequences of early HI brain injury (Hill and Fitch, 2012) but also to socio-economic and neonatal variables (Månsson et al., 2015). In spite of these sex differences, most studies involving HI still demonstrate usage of male and female animals indistinctly (i.e., Chou et al., 2001; Ten et al., 2003; Lubics et al., 2005; Spandou et al., 2005; Ikeda et al., 2006; Pazos et al., 2012).

On the other hand, developmental psychobiology and neuroscience have pointed out ontogeny as a singular window of brain vulnerability but also plasticity, where early-life paradigms involving exposure to distinct stimuli during the 1st weeks of life are shown to be critical for short- and long-lasting modeling of the brain structure and function (Levine, 1957; Levine and Broadhurst, 1963; Levine et al., 1967). In rodents, early postnatal stimulation [neonatal handling (NH) in its most frequent form] consisting of brief maternal separation with/without tactile stimulation, prompts profound and long-lasting effects of anxiety and stress responses, novelty-seeking, learning and memory through different epigenetic neurobiological mechanisms (revised by Fernández-Teruel et al., 2002). More importantly, compelling effects of this intervention also include the rescue of perinatal brain insults due to stress, malnutrition or alcohol exposure (revised by Raineki et al., 2014). In the case of brain injury induced by HI, prevention of hippocampal damage (Rodrigues et al., 2004) and improvement of learning (Chou et al., 2001) were elicited in rats by maternal separation and tactile stimulation starting at PND8. Beneficial effects of tactile sensory stimulation have been attributed to the fact that neural pathways from skin to the CNS mature before other sensory systems (Montagu, 1953).

Therefore, the aim of the current research work was to do a longitudinal study of the behavioral and functional impact of HI brain injury in male and female mice, at weaning and at adulthood. We also aimed to assess the effects of NH used as a protective sensory intervention. For this purpose, we evaluated the short- and long-term effects of a neonatal HI insult in the behavioral profile of gold-standard C57BL/6 mice strain at PND7, an age modeling full-term babies. At the same time, a group of animals was used to assess the potential preventive effects of NH administered during the ontogenic development of the pups, from PND1 to weaning (PND21), mimicking an intervention started in pre-term babies and lasting all through their childhood. For the behavioral and functional screening of the pups at weaning (PND23) and their long-term outcomes (adulthood, PND70) we used a series of tests to evaluate four dimensional areas: somatic development and sensorimotor integration, exploratory behavior, emotionality and anxiety-like behaviors, and cognition. Finally brain pathology was measured by means of brain injury score to evaluate the neurologic impact of the risk/protection events in different neuroanatomical regions at 90 days of age (PND90). This will allow us to find the functional correlates.

## Materials and Methods

### Animals

Sixty-six C57BL/6 mice were bred and maintained in macrolon cages (35 cm × 35 cm × 25 cm) under standard laboratory conditions (food and water *ad libitum*, 22°C ± 2°C, 50%–70% relative humidity, 12 h light:dark cycle, lights on at 08:00 h).

### Experimental Design

**Figure 1:**
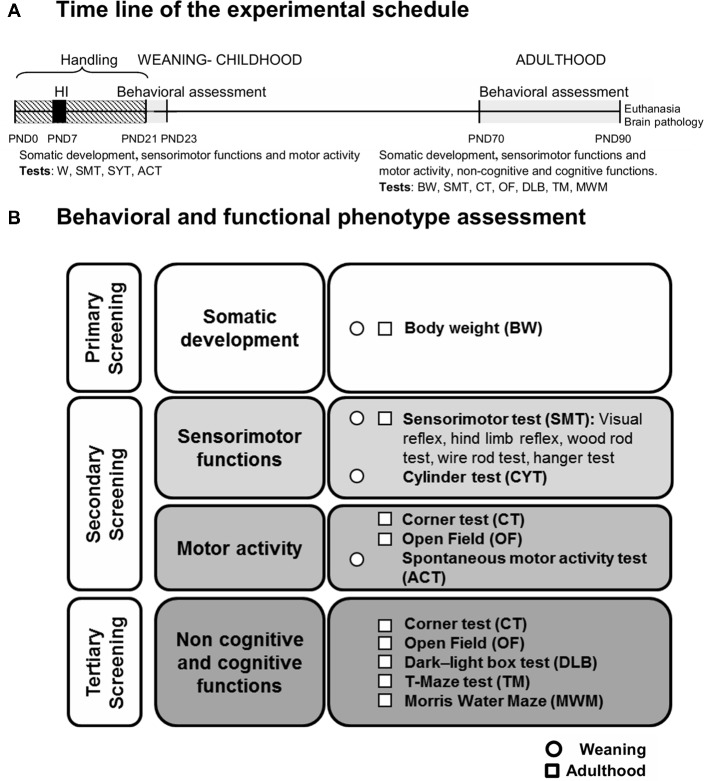
**(A)** Time line of the experimental schedule. **(B)** Behavioral and functional phenotype assessment: a three-stage protocol for behavioral and functional phenotype assessment evaluated the somatic development (primary screening), sensorimotor functions and motor activity (secondary screening) as well as non-cognitive and cognitive functions (tertiary screening). The tests used were the following: somatic development (weight), sensorimotor functions (visual and hind limb reflexes, rod test and hanger test), locomotor [activity test (ACT) and open-field (OF) test] as well as non-cognitive and cognitive functions-emotional and anxiety-like behaviors [corner, OF and dark-light box (DLB) tests]; learning and memory [T-maze test (TM) and Morris Water Maze (MWM)]. The behavioral assessment was performed at weaning and/or adulthood.

A longitudinal study with factorial design HI × NH × S was made to evaluate the functional impact of HI, the effects of NH in sham and HI animals, and the factors sex and age. Age factor was studied to evaluate the long-term effects, from weaning at PND23 to adulthood (PND70), of the risk/protective interventions studied. The total 66 mice came from 12 L with an average of 5.50 + 0.45 pups. Litters were randomly distributed by treatments and gender in 8 different experimental groups (*n* = 7–11, Figure 1A). Two animals (1 male/sham, 1 female/hypoxia) died from PND23 to PND90, and were therefore excluded of the statistical analysis.

### Hypoxia/Ischemia

HI brain damage occurred at PND7 by permanent left carotid occlusion and exposure to hypoxia as previously described (Sheldon et al., 2001). Briefly, a midline ventral skin incision was made under isoflurane anesthesia (4.5% v/v for induction and 2.5% v/v for maintenance, and 0.6 L/min of O_2_); the left carotid artery was exposed and sutured with a 8/0 silk surgical suture. After surgery, pups were returned to their dam for at least 1.5 h to recover. Later, litters were placed for 55 min in a hypoxic chamber containing 8% of oxygen balanced with nitrogen, with controlled humidity and temperature maintained at 37°C.

### Handling

NH was administered from PNDs 1 to 21 (Fernández-Teruel et al., 1991). The first daily session, administered in the morning (9:30 a.m.), consisted of first removing the mother from the litter, and then weighing the pups and placing them gently and individually in plastic cages (35 cm × 15 cm × 25 cm) lined with soft article towel. After 4 min in this situation, each pup was individually (and gently) handled and stroked thrice with the thumb on the dorsal surface (rostro-caudal direction) for 3–4 s and returned to the same cage for the remaining 4 min. At the end of the 8-min period, each pup was gently handled for another 3–4 s, stroked again and then returned to its home cage. When all the pups from 1 L were back in their home cage, the mother was returned to it. The same procedure (without weighing the animals) was conducted in the afternoon (2nd time; approximately at 4:30 p.m.). NH finished at PND 21. Weaning was done at PND 21, after finishing the last NH session. Non-handled groups were left undisturbed, except for regular cage cleaning once a week, until weaning.

### Behavioral and Functional Assessments

Animals were assessed in a longitudinal design, at weaning (PND23) and adulthood (PND70 or 70-days-old) as summarized in the time line shown in Figure 1. A three-stage protocol (Figure 1B) for behavioral and functional phenotype assessment (Giménez-Llort et al., 2002) evaluated the somatic development (primary screening), sensorimotor functions and motor activity (secondary screening) as well as non-cognitive and cognitive functions (tertiary screening). The tests used were as follows: somatic development (weight), sensorimotor functions [visual and hind limb reflexes, rod test and hanger test, cylinder test (CYT)], locomotor [activity (ACT) and open-field (OF) test] as well as non-cognitive and cognitive functions-emotional and anxiety-like behaviors [corner, OF and dark-light box tests (DLB)]; learning and memory (TM test and MWM). All the apparatus were thoroughly cleaned, with 5% ethanol, and dried between trials/animals.

### Body Weight (BW)

Body Weight (BW) was monitored at weaning (PND23), adulthood (PND70) and at the end point (PND90).

### Sensorimotor Functions (SMT)

The physical condition of the mice was evaluated by their BW and performance in sensorimotor tasks. Visual reflex and hind limbs extension reflex were measured three times by holding the animal by its tail and slowly lowering it toward a black surface. Complete extension of the forelimbs towards the surface (visual reflex) or the extension of hind limbs were considered a positive response. Motor coordination and equilibrium were assessed twice (20-s trials) in two consecutive rod tasks of increasing difficulty. The distance covered and the latency to fall off a 1.3 cm wide wooden wire rod and a 1 cm diameter metal wire rod (both, 1 m long) were recorded. The hanger test was used to measure prehensility or grasping and motor coordination by the distance covered and the number of elements of support and the latency to fall. The animal was allowed to cling with its forepaws from the middle of a horizontal wire (2 mm diameter, 40 cm length, divided into eight 5 cm segments) for two trials of 5 s. A third trial of 60 s was used to complement these measures with that of muscle strength or resistance. All the apparatus were suspended 40 cm above a padded table.

### Cylinder Test (CYT)

The CYT was used to assess forelimb asymmetry (Schallert et al., 2000). Animals were individually placed in a Plexiglas transparent cylinder (10 cm diameter, 12 cm height). Each animal was video-recorded for 5 min. Initial forepaw (left/right/both) placement of each weight-bearing full rear was recorded. The asymmetry score that reflected the preference of the unimpaired (left) limb was calculated according to the following formula:[(number of left contacts + $\frac{1}{2}$ both contacts)/number of (left + right + both contacts)]×100; where 50% indicates an animal that explores symmetrically with both limbs, higher scores (>50%) indicate a greater reliance on the ipsilateral limb, and lower scores (<50%) indicate a greater reliance on the contralateral limb. We also measured the number and the total amount of grooming time, the latency of “hoppy” or “pop-corn behavior” described by Wahlsten (1974) as vigorous jumping shown in pups, and the number of full rears performed in the 5 min.

### Spontaneous Motor Activity Test (ACT)

The mice were individually tested in a multicage activity meter system (four home cages −35 cm × 35 cm × 25 cm—simultaneously, Sensor Unit PANLAB 0603, Panlab, S.L., Barcelona, Spain) set to measure horizontal and vertical spontaneous motor activity during 30 min. Animals were individually tested in a standard (but novel and clean) home cage containing a small amount of clean sawdust on the floor.

### Corner Test (CT) and Open Field Test (OF)

Neophobia was assessed in the corner test (CT) for 30 s. Animals were individually placed in the center of a clean standard home cage, filled with wood save bedding. Latency of the first rearing, number of corners visited and of vertical displacements (rearings) were recorded. Immediately after, exploratory and anxiety-like behaviors were measured during 5 min in a white open-field (homemade, wooden, 55 × 55 × 25 cm) under 20 lux light conditions. Mice were individually placed in the center of the arena. Horizontal (crossings, 10 × 10 cm) and vertical (rearings) activities were recorded for each minute of the test.

### Dark–Light Box Test (DLB)

Anxiety and risk assessment were measured for 5 min after introducing the animals into the dark compartment of the DLB (Panlab, S.L., Barcelona, Spain). The apparatus consisted of two compartments (black, 27 cm × 18 cm × 27 cm, white, 27 cm × 27 cm × 27 cm illuminated by a red 20 W bulb) connected by an opening (7 cm × 7 cm). The experimental room was kept in darkness (without illumination). Mice were introduced into the black compartment and observed for 5 min. Total number of entries (all four paws), and time spent in the white-illuminated compartment were recorded. Latency to enter, time spent and number of entries into the lit compartment, the number of stretch attendances and self-groomings were recorded.

### T-Maze Test (TM)

Exploratory activity, anxiety, working and short-term memory were assessed in an enclosed TM (woodwork; short arm: 40 × 15 × 30; goal arms: 30 × 15 × 30 cm), three trials 15 min apart, in a day. Each trial involved one forced and one free choice. Mice were placed inside the short arm of the maze and the latency to reach the intersection and the time elapsed until mice completed 20 s in the forced arm were recorded. Fifteen seconds later, mice were allowed to explore the maze in a free choice trial where both arms were accessible. The arm chosen was recorded and considered an error if it was not different of that in the forced choice.

### Morris Water Maze (MWM)

Animals were tested for spatial learning and memory in three paradigms in the MWM test consisting of 1 day of cue learning and 4 days of place learning for spatial reference memory, followed by one probe trial. Mice were trained to locate a hidden platform (7 cm diameter, 1 cm below the water surface) in a circular pool for mice (Intex Recreation Corp., Long Beach, CA, USA; 91 cm diameter, 40 cm height, 25°C opaque water), located in a completely black painted 6 m^2^ test room. Mice failing to find the platform were placed on it for 10 s, the same period as the successful animals. The protocol (Giménez-Llort et al., 2007) was used as follows: 1 day of cue learning, 4 days of place learning followed by a probe trial.

#### Cue Learning With a Visible Platform

On the first day, the animals were tested for the cue learning of a visual platform consisting of four trials in 1 day. In each trial, the mouse was gently released (facing the wall) from one randomly selected starting point (E or W) and allowed to swim until it escaped onto the platform, elevated 1 cm above the water level in the *N* position and indicated by a visible striped flag (5.3 × 8.3 × 15 cm). Extra maze cues were absent in the black painted walls of the room.

#### Place Learning With a Hidden Platform

On the following day, the place learning task consisted of three trial sessions per day for 4 days with trials spaced 30 min apart. The mouse was gently released (facing the wall) from one randomly selected starting point (E or W, as these are equidistant from the target) and allowed to swim until escaped onto the hidden platform, which was now located in the middle of the S quadrant. Mice that failed to find the platform within 60 s were placed on it for 10 s, the same period as was allowed for the successful animals. White geometric figures, one hung on each wall of the room, were used as external visual clues.

#### Removal

Two hours after the last trial of the place learning task, the platform was removed from the maze and the mice performed a probe trial of 60 s to evaluate their spatial memory for the platform position.

#### Analyses

Behavior was evaluated by both direct observation and analysis of videotape-recorded images. Variables of time (escape latency, quadrant preference), distance covered, and swimming speed were analyzed in all the trials of the tasks. The escape latency was readily measured with a stopwatch by an observer who was unaware of the experimental group, and was confirmed during the subsequent video-tracking analysis. A video camera placed above the water maze recorded the animal’s behavior and thereafter an automated system (Smart, Panlab S.L., Barcelona, Spain) enabled computerized measurement of the distance traveled by the animal during the trials. The swimming speed (cm/s) of the mice during each trial was calculated. In the probe trial, the time spent in each of the four quadrants, the distance traveled along them, and the number of crossings over the removed platform position (annulus crossings) were also measured retrospectively by means of the automated video-tracking analysis.

### Neuropathological Analysis

Brain damage was analyzed by histological analysis at PND90 (Shrivastava et al., 2012). Mice were i.p. anesthetized (ketamine and xylazine 80/10 mg/Kg) and perfused using 4% paraformaldehyde in phosphate buffer (PB, pH 7.4). Subsequently, brains were postfixed for 4 h in the same fixative, cryoprotected in 30% sucrose, frozen with dry CO_2_, and finally stored at −80°C until use. Brains were serially cut in a cryostat (Leica CM3050 S) in 30 μm thick sections and stored in −20°C mounted on Flex IHC slides (Dako). To determine the injury score, slides were processed for Nissl staining. One series of parallel sections from each animal (6–10 mice/survival time) was air dried at room temperature for an hour, rinsed and incubated with Nissl solution (0.1% toluidine blue in walpole buffer 0.2 M and pH 4.5) at room temperature for 3 min and washed with distilled water. Sections were dehydrated, cleared in xylene, and coverslipped with DPX.

### Statistics

Results are expressed as means ± SEM. Repeated measures ANOVA (RMA) with a 2 × 2 × 2 HI × NH × S factorial analysis with HI, NH and S (sex) as main factors and within subjects A (Age) or T (Time course) analysis was used followed by *post hoc* tests. Two independent measures were analyzed with Student’s *t*-test, while those obtained for the same animals at PND23 and PND70 were analyzed with paired *t*-test. Behavioral correlations with brain damage were analyzed with Pearson’s correlation. *P* < 0.05 was considered statistically significant.

## Results

### Short and Long Effects of HI and Their Counteraction by Handling

#### Neuropathological Analysis

**Figure 2:**
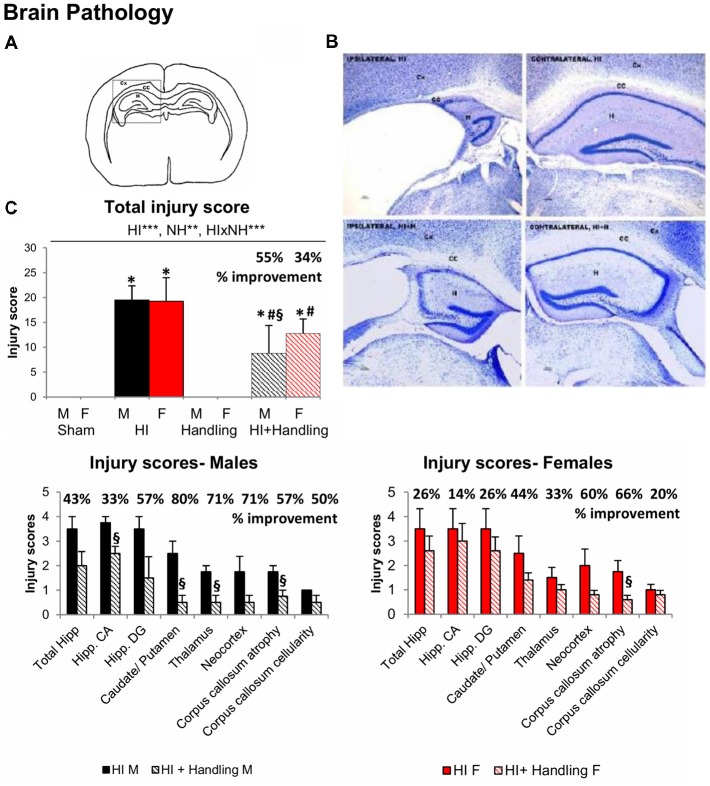
Neuropathological analysis after hypoxic/ischemic (HI). **(A)** Drawing modified in Adobe Photoshop CS showing brain areas analyzed for quantification of brain damage in the ipsilateral side. CX, cortex; CC, corpus callosum; H, hippocampus. **(B)** Nissl staining showing HI effects on the cortex, hippocampus and CC of the contralateral (right side of the panel) and ipsilateral (left side of the panel) hemisphere 90 days after hypoxia. **(C)** Graphs show the changes in the total injury score along with the injury score in different regions analyzed. HI males and females presented higher injury scores than sham and HI + NH mice when we analyzed total injury score, the last ones were also different from sham. When we measured the injury scores in the different brain regions, we observed that HI + NH males were different from HI in most regions; however, in females, the significant differences only appeared when we measured the CC atrophy. Results are presented as mean ± SEM. Statistics: repeated measures analyses of variance (RMA), two-way ANOVA: “HI” Hypoxia effect; “NH” handling effect; “S” sex effect; “A” age effect *P < 0.05, **P < 0.01, and ***P < 0.001. Student *t*-test. *P < 0.05 vs. sham of the same sex; ^#^*P* < 0.05 vs. handling of the same sex; ^§^*P* < 0.05 vs. hypoxia of the same sex.

HI and NH effects and HI × NH interaction were found in the neuropathological analysis (Figure 2). No sex effects were detected at the level of brain injury induced, as shown by the injury scores and histological appearance (only males shown). HI animals presented high injury scores as compared to null values in the two control groups, sham and handled. HI + NH animals were not exempt of injury (vs. sham or handling, all *F*_(1,65)_ > 7.9463, *P* < 0.009), but the total score and that at the different neuroanatomical areas was significantly decreased as compared to HI animals. When we measured the injury scores in the different brain regions, we observed that HI + NH males were different from HI in most regions; however, in females, the significant differences only appeared when we measured neocortex and the corpus callosum (CC) atrophy (Student’s *t*-test, all *P* < 0.04).

### Somatic Development (Primary Screening)

#### Body Weight (BW) PND23–PND70–PND90

**Figure 3:**
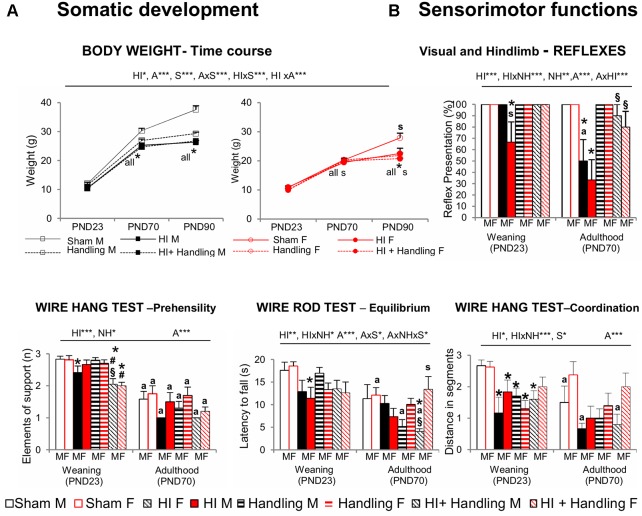
**(A)** Somatic development [postnatal day (PND)23–PND90] and **(B)** sensorimotor functions (PND23–PND70). Short- and long-effects of HI in males and females C57BL6 and effects of neonatal handling (NH). Results are presented as mean ± SEM. HI animals presented slower weight gain, with male being more sensitive than female; handling could not reverse the hypoxia’s effect. At sensorimotor level, HI impaired visual and hindlimb reflexes, which were first shown in females (PND23). Equilibrium, prehensility and coordination were also affected. Handling modulated the impaired reflex responses and an interaction with HI was also seen in other sensorimotor functions. Statistics: RMA, two-way ANOVA: “HI” Hypoxia effect; “NH” handling effect; “S” sex effect; “A” age effect *P < 0.05, **P < 0.01, and ***P < 0.001, followed by Duncan’s *post hoc* test. *P < 0.05 vs. sham of the same sex; ^#^*P* < 0.05 vs. handling of the same sex; ^§^*P* < 0.05 vs. hypoxia of the same sex; ^s^*P* < 0.05 female vs. male of the same treatment. Paired *t*-test comparison day 23 vs. day 70, ^***a***^*P* < 0.05.

HI resulted in slower weight gain (Figure 3A) that was sex-dependent and worsened with age (both *P* < 0.001). Less weight gain was apparent in males since PND70, while in female the differences were observed later, at PND90 (S × A, *P* < 0.001). Handling was not able to reverse this HI effect.

### Sensorimotor Functions and Motor Activity (Secondary Screening)

#### Sensorimotor Functions (SMT)-PND23-PND70

At the sensorimotor level (Figure 3B), HI females showed impairment of visual and hind limb extension reflexes, even at PND23 and worsening at PND70, which was the same for males of this age (all, *P* < 0.05). Prehensility, equilibrium and coordination measured in the wire rod and hanger tests were also found to be impaired and were in this order of severity (*P* < 0.001, *P* < 0.01 and *P* < 0.05, respectively). Handling was able to modulate the HI-induced impairment in reflex responses and HI × NH interaction effects were observed in the coordination and equilibrium. However prehensility (elements of supports when holding from a hanger) was not improved by handling.

#### Cylinder Test (CYT)—PND23

**Figure 4:**
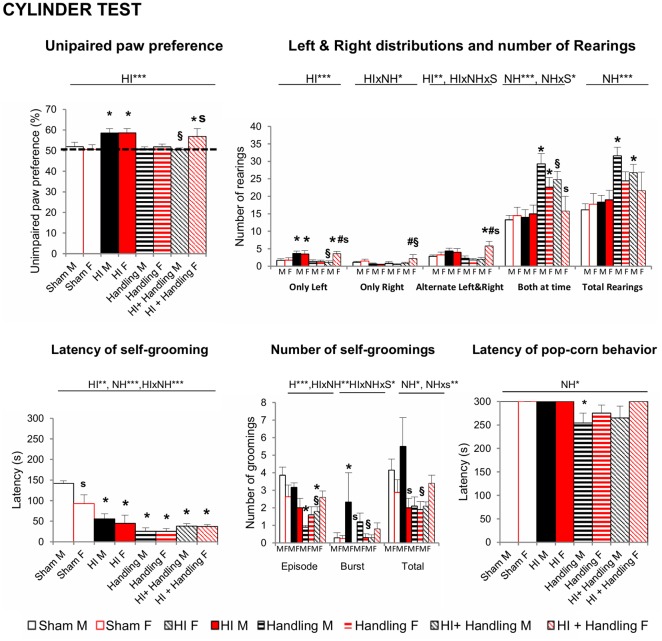
Cylinder Test (CYT): short- and long-effects of HI in males and females C57BL6 and effects of NH. HI of both genders and HI + NH females presented higher paw preference scores (>50%) which indicate a greater reliance on the ipsilesional limb. Moreover, these groups presented a greater number of left rearings and H + NH female also presented more alternate left and right contacts. When we measured the total rearings performed, handling and HI + NH males presented the higher scores. Latency of first self-grooming behavior showed a clear sex-dependent effect, with females being faster in the expression of this emotivity-related behavior. All the other groups, showed fast elicitation of it, too. However, in the male sex, the total number of episodes made a difference between HI-animals and those with handling or HI+NH. Latency of pop-corn behavior was shorter in groups with handling, reaching statistical significance in handled males. Statistics: RMA, two-way ANOVA: “HI” Hypoxia effect; “NH” handling effect; “S” sex effect; “A” age effect *P < 0.05, **P < 0.01, and ***P < 0.001, followed by Duncan’s *post hoc* test. *P < 0.05 vs. sham of the same sex; ^#^*p* < 0.05 vs. handling of the same sex; ^§^*P* < 0.05 vs. hypoxia of the same sex; ^s^*P* < 0.05 female vs. male of the same treatment.

In the CYT (Figure 4) HI increased the incidence of unimpaired paw preference in both males and females, (*P* < 0.001) and handling was able to reserve this effect, but only in males (*P* < 0.05). However, the detailed analysis of the use of the paws (used alone, alternatively, both at a time and the total number of vertical rearings) indicated that NH exerted beneficial effects also in females, as they showed an increased number of rearings using the impaired right arm, and were able to alternate both arms (*F’*s_(1,65)_ > 3.168, *P* < 0.05). Latency of first self-grooming behavior showed a clear sex-dependent effect, with females being faster in the expression of this emotivity-related behavior. All the other groups, showed fast elicitation of it, too. However, in the male sex, the total number of episodes made a difference between HI-animals and those with NH or HI+NH. It is also important to note the appearance of burst, attempts to initiate the self-grooming, as a characteristic of HI-males, that was restored to normal levels in HI+NH (*F’*s_(1,65)_ > 2.37, *P* < 0.05). Latency of pop-corn behavior was shorter in groups with NH, reaching statistical significance in NH males (*P* < 0.05).

#### Spontaneous Motor Activity Test (ACT)–PND23

**Figure 5:**
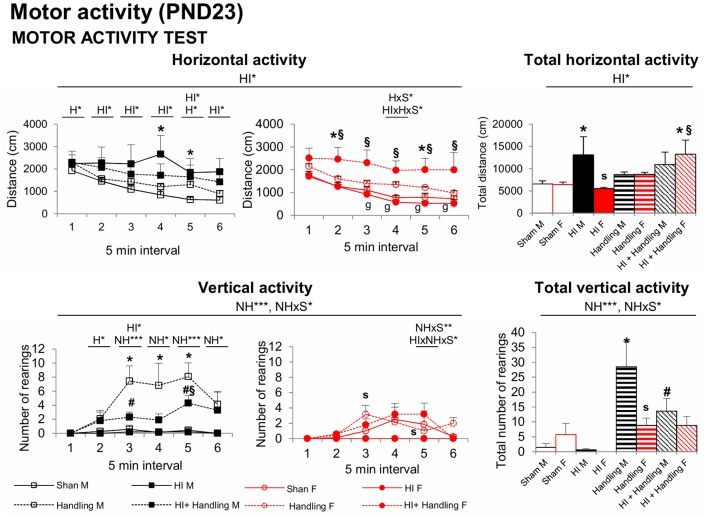
Spontaneous motor ACT: short- and long-effects of HI in males and females C57BL6 and effects of NH. Motor activity (PND23). Horizontal activity in ACT was increased in HI males and HI + NH females; however, the vertical activity was higher in handling males. Statistics: RMA, two-way ANOVA: “HI” Hypoxia effect; “NH” handling effect; “S” sex effect; “A” age effect *P < 0.05, **P < 0.01, and ***P < 0.001, followed by Duncan’s *post hoc* test. *P < 0.05 vs. sham of the same sex; ^#^*P* < 0.05 vs. handling of the same sex; ^§^*P* < 0.05 vs. hypoxia of the same sex; ^s^*P* < 0.05 female vs. male of the same treatment.

At PND23 in the spontaneous motor ACT, the time course and total motor activity counts recorded in a 30 min period indicate that HI increased the horizontal component of activity in males (vs. sham, *P* < 0.05) and that NH counteracted this effect (Figure 5). However, HI+NH females presented an increased activity. Like in the CYT, NH males presented a significant increase in rearings in comparison to sham, HI female and HI+NH male (*P* < 0.05).

### Non-Cognitive and Cognitive Functions (Tertiary Screening)–PND70–PND90

#### Corner Test (CT) and Open Field (OF)

**Figure 6:**
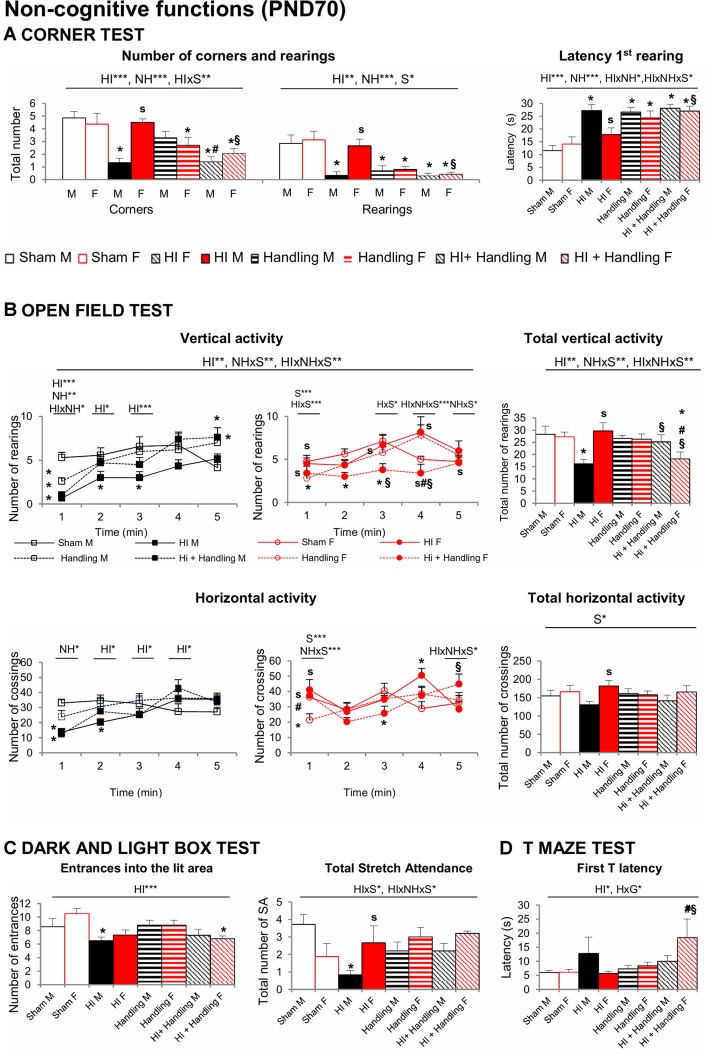
Corner test (CT). OF. DLB. TM: short- and long-effects of HI in males and females C57BL6 and effects of NH. Non-cognitive functions were evaluated during PND70–PND90. Results are presented as mean ± SEM. **(A)** The neophobia in the CT was increased in hypoxia males and in both handling and HI + NH males and females. HI induced anxiety-like behavior in the OF **(B)** that lead to reduced exploratory activity with males being mostly affected. The anxious profile could be also observed in several variables measured by DL **(C)** were HI males and HI + NH females performed fewer entrances into the lit area. Moreover, HI males presented also fewer number of stretch attendance. Finally, in the TM **(D)** the highest values in the first T-latency were spent by HI + NH female, although there’s no significant difference, HI male also spent more time to reach the intersection. Statistics: RMA, two-way ANOVA: “HI” Hypoxia effect; “NH” handling effect; “S” sex effect; “A” age effect *P < 0.05, **P < 0.01, and ***P < 0.001, followed by Duncan’s *post hoc* test. *P < 0.05 vs. sham of the same sex; ^#^*P* < 0.05 vs. handling of the same sex; ^§^*P* < 0.05 vs. hypoxia of the same sex; ^s^*P* < 0.05 female vs. male of the same treatment.

Significant effects of HI, NH and S were found in the CT (Figure 6A). Horizontal activity measured by numbers of corners visited was reduced in HI and HI + NH mice. Vertical activity was also influenced by hypoxia and handling, with these animals showing higher latencies to perform a first rearing and a reduction in the total number of rearings (all *F’*s_(1,65)_ > 5.457, *P* < 0.05). HI males were more neophobic than sham and HI females; NH and HI+NH animals of both genders were also more neophobic than their respective shams.

In the OF Test, the ethogram (sequence of behavioral events) was faster in sham, handled males and females and only in HI and HI+NH females reaching the statistical significance in most of the latencies studied (all *P* < 0.05). Accordingly, the 1st minute of test was the most sensitive showing HI, NH and G differences with HI × NH, HI × S and NH × S interactions (*F’*s_(1,65)_ > 5.038, *P* < 0.05), especially in vertical activity. Regarding the locomotor activity, HI effects with NH × S and HI × NH × S interactions were showed when we study the total vertical activity (*F’*s_(1,65)_ > 7.401, *P* < 0.05). HI males performed less rearings than sham, HI+NH males and HI females while HI+NH females performed lesser rearings than sham, HI and NH females (*P* < 0.05). Moreover, in total horizontal activity, HI males performed lower number of crossing than HI females (*P* < 0.05) No differences in self-grooming or defecation were recorded (Figure 6B).

#### Dark-Light Box Test (DLB)

HI effect was observed when we measured the number of entrances into the lit area (Figure 6C), where HI males and HI+NH females exhibited reduced number of entries. Stretch attendance activity reflected HIxS and HIxNHxS interactions (all *F’*s_(1,65)_ > 4.154, *P* < 0.05). HI males also presented a reduced number of stretch attendance in comparison with sham and HI females. No more significant anxiety changes were detected in the other variables.

#### T-Maze Test (TM)

No changes in working memory were apparent in the TM. Differences in the first latency to reach the intersection (Figure 6D) were found with significant HI effect and NHxS interactions (all *F’*s_(1,65)_ = 5.326, *P* < 0.05). The highest latency to reach the intersection point of the TM was observed in HI+NH females as compared to other counterparts. Moreover, HI males presented a higher first T-latency in comparison with sham, although there is no significant difference. HI male also spent more time reaching the intersection.

#### Morris Water Maze (MWM)

**Figure 7:**
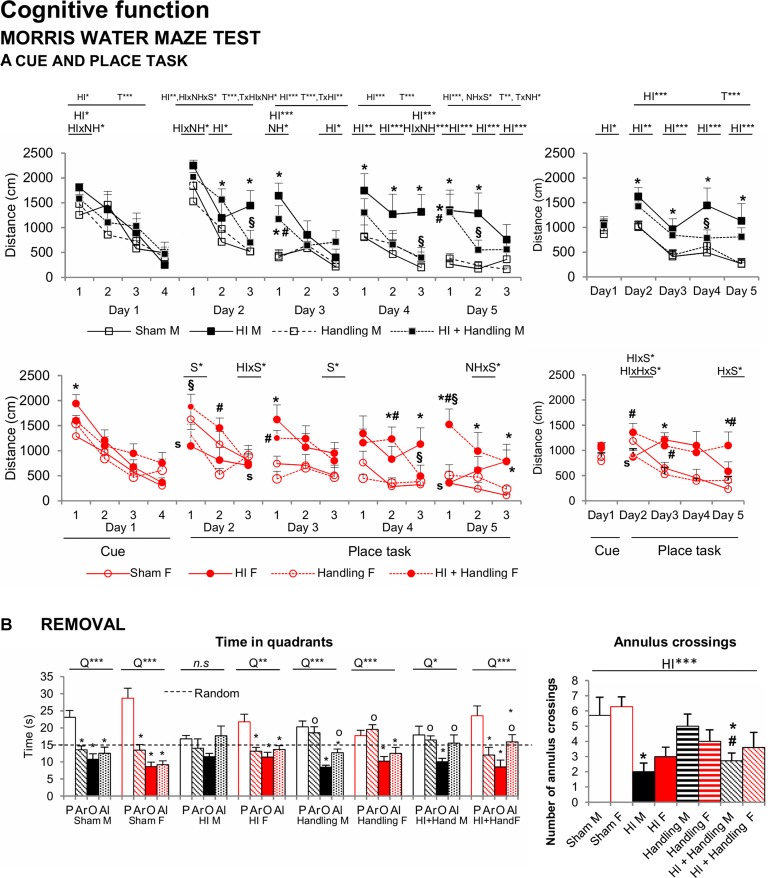
MWM: short- and long-effects of HI in males and females C57BL/6 and effects of NH. Cognitive functions were evaluated during PND70–PND90. Results are presented as mean ± SEM. Mean distance to reach the platform in the cue and place tasks for spatial learning. All days presented time effect. **(A)** Cognitive deficits were observed in the MWM in both sexes with reduced total learning capacities (acquisition of the task) and worse short and long-term learning. In HI mice a reduction of the mean distance covered to find the platform could be recorded in both “trial-by-trial” and “day-by-day”, especially in place tasks learning and were mainly important in HI males. Memory in HI males was more clearly affected than females in the MWM, however it was modulated by handling **(B)** In the probe trial for short-term memory, hypoxia males did not distinguish between the trained quadrant (P) and the adjacent (Ar, Al) or the opposed one (O) presenting a random swimming while handling (male and female) and HI + NH male presented a scanning swimming. Finally sham (male and female), HI and HI + NH female presented a focal searching swimming due they distinguish between the trained quadrant and the rest of the quadrants. The number of annulus crossings was also fewer in HI and HI + NH males. Statistics: Duncan’s *post hoc* test. *P < 0.05 vs. sham of the same sex; ^#^*P* < 0.05 vs. handling of the same sex; ^§^*P* < 0.05 vs. hypoxia of the same sex; ^s^*P* < 0.05 female vs. male of the same treatment. Preference for the trained quadrant (P) as compared to adjacent right (Ar) and left (Al) or Opposite (O) quadrants in the probe trial. ANOVA, “Q” preference for trained quadrant. *P < 0.05, **P < 0.01, and ***P < 0.001; followed by Duncan’s *post hoc* test *P < 0.05 vs. P quadrant, ^o^*P* < 0.05 vs. O quadrant. n.s. non-significant statistical differencs is found.

Figure 7A illustrates the “day-by-day” (left panel) and “trial-by-trial” (right panel) acquisition curves. All days presented the temporal effect (all *F’*s_(1,65)_ = 9.319, *P* < 0.001), especially in place task learning, when the cue was removed and the platform was hidden, animals exhibited different acquisition curve. The HI and HI + NH animals found the hidden platform slower along the 4 days of the test as showed by a longer distance covered to find the platform in comparison to sham mice (HI and S effects; HI × NH, HI × S and HI × NH × S interactions, *F*_(1,65)_ > 3.908, *P* < 0.05). Memory in HI males was more clearly affected than females in the probe trial of the MWM (Figure 7B) for short-term memory. HI males did not distinguish between the trained quadrant (P) and the adjacent (Ar, Al) or the opposed ones (O) presenting a random swimming. In contrast handled (male and females) and HI+NH males presented a scanning swimming. Finally, sham (male and female), HI and HI+NH females presented a focal searching swimming as they distinguished between the trained quadrant and the rest of the quadrants (Lang et al., 2003; all *P* < 0.05). Although we observed that this memory effect in males was modulated by handling, HI and HI + NH males presented a worse performance when we analyzed the annulus crossing in comparison to sham (HI effect *F*_(1,65)_ = 17.047, *P* < 0.001, *post hoc* Duncan’s test, all *P* < 0.05).

#### Correlations Analysis

**Figure 8:**
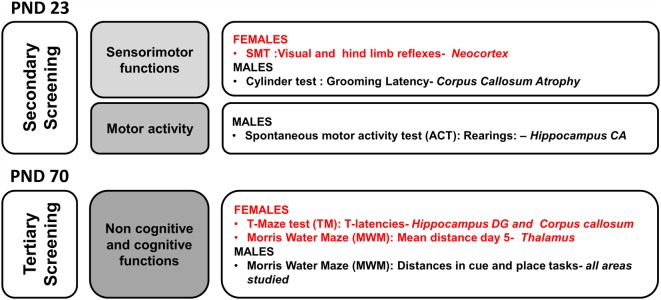
Neuroanatomical correlates of short- and long-term behavioral effects of HI in males and females C57BL/6 and those of NH. The graphical abstract summarizes the strongest neuroanatomical-behavioral correlations (*P* < 0.01) found in males (55 variables) and females (17 variables). This distribution, indicates that in spite of similar injury score, the NH was more effective restoring functions in males and that they were those related to the cognitive domains. In contrast, the domain of non-cognitive function was the more benefited in HI + NH females.

**Figure 9:**
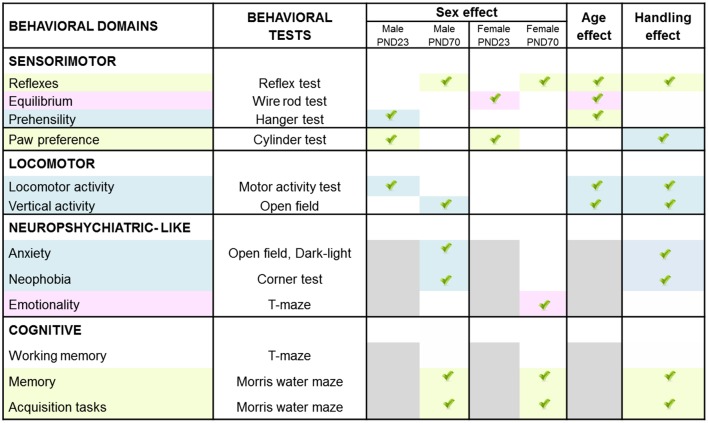
Graphical abstract of sex- age- and task-dependent behavior impact of HI brain injury and its modulation by handling.

In a matrix of 175 × 9 (behavioral × injuries score) variables studied, we performed a meaningful correlation analysis (see Figure 8) between those variables where HI showed a deleterious effect and/or handling an effect (see Figure 9). Neonatal handled and sham animals were excluded from this analysis, since injury score was null. As detailed in Figure 8, the strongest correlations (*P* < 0.01) were found in 55 cases in male variables as compared to 17 cases in females. This distribution indicates that in spite of a similar injury score, the NH was more effective in restoring functions in males and within those related to the cognitive domains. In contrast, the domain of non-cognitive function was improved more in HI + NH females.

## Discussion

The present work addresses the short and long-term behavioral and functional impact of neonatal HI brain injury in a term (PND7) mice model using a longitudinal approach and taking into consideration sexual dimorphism. It also provides evidence of a remarkable neuropathological protection elicited by NH. This tactile and proprioceptive sensory stimulation was administered to mice from PND1, a temporal frame modeling premature children, when the human immature brain is at increased risk of HI injury, mostly in males. NH not only ameliorated the behavioral outcomes and functional capacities but also showed differences in a sex- age- and task-selective manner. Several levels of study were considered from behavior to neuropathology, including key areas of clinical interest such as BW, sensorimotor function, physical/motor activity, emotionality, cognitive function and finally brain pathology in different neuroanatomical regions. The evaluation of these risk/protective events studied at weaning and adulthood indicates that benefits observed in the developmental outcome also result in long-term sustained effects, which contributes to their translational interest. Besides, the results indicate that even with similar HI-induced brain injury scores, interaction effects with sex are important to be taken into consideration when assessing the outcome of preventive and/or therapeutic strategies.

In contrast to most studies describing detailed neuropathological changes in the rat model, here we provide the neuropathological tissue damage at 90 days, and the neuroanatomical distribution in both male and female C57BL/6 mice strains. In agreement with a devastating early PBI insult, the histopathological analysis of HI brains provided evidence of the severity of the experimentally induced brain injury, and the histopathological protection conferred by NH intervention. PBI is considered as one of the major contributors to perinatal morbidity and mortality. Here, the mean index of postnatal mouse mortality due to surgery or hypoxia was 19.31%, with 18.46% for males and 20.00% for females, showing no statistical differences between sexes.

High variability in size and severity of the infarct between animals is a significant drawback of the HI experimental model (Vannucci and Vannucci, 2005; Millar et al., 2017). It seems that sex, severity, time of injury or even the brain lateralization of the lesion can significantly affect the outcome of Rice-Vannuci model (Lubics et al., 2005; Sanches et al., 2013b, 2015). In this regard, it is noticeable that, in the present work, the HI-procedure induced homogeneous total injury scores and neuroanatomical distribution in both sexes. Most importantly, this allowed us to highlight the capacity of NH to reduce the injury score in most of the areas, albeit not all of them reached statistical significance.

In contrast to other early life interventions studied in the literature, we unveil for the first time a relevant sex-dependent improvement of NH on HI, with HI + NH males being more responsive than females. This is important to note since sex differences have been considered in recent years. It has been reported that male infants are more vulnerable to perinatal insult than female infants, and they also suffer more long-term cognitive deficits. Males showed increased risk of development disorders, including speech and language, autism, learning disabilities and cerebral palsy compared to females (Donders and Hoffman, 2002; Rutter et al., 2003; Marlow et al., 2005; Tioseco et al., 2006; Hill and Fitch, 2012). Some authors also refer that being “male” has been identified as a universal risk factor for the incidence of neonatal stroke as well as developmental delays (Elsmén et al., 2004; Peacock et al., 2012; Månsson et al., 2015). Moreover, after pediatric traumatic brain injury, girls demonstrate a significantly better outcome in tests of learning and memory (Donders and Hoffman, 2002; Hurn et al., 2005). In our present work, HI males showed a worse performance in the memory test as compared to HI females and the improvement induced by NH in HI males was supported by better levels of hippocampal preservation.

Behavioral and functional phenotype assessment was performed using a three-stage protocol (Giménez-Llort et al., 2002). The somatic development was evaluated as primary screening, and BW was used for that. Feeding dysfunction and nutritional problems has been associated with poor growth and health status in children with cerebral palsy and neurological impairment (i.e., Reilly et al., 1996; Sullivan et al., 2000; Fung et al., 2002). Experimentally, the few number of studies addressing this issue has shown lower daily weights in HI rats (Andiné et al., 1990; Balduini et al., 2001; Lubics et al., 2005; Girard et al., 2012). Here we found differences in growth rate of HI mice that worsened with age and were long-lasting. The results also indicate that in males sex BW was more sensitive to the impact of HI than in females, who exhibited lower weight later in adulthood. This was in agreement with the sex-dependent vulnerability that we found in the behavioral outcome.

The secondary screening evaluated the sensorimotor functions and motor activity. Strength, motor coordination and several reflex responses (righting, geotaxis, gait and cliff aversion) were reported as impaired after HI insult in rats and mice (Ten et al., 2003; Fan et al., 2005; Lubics et al., 2005; Karalis et al., 2011). Here, other sensorimotor tasks such as the visual and extension reflexes, prehensility or grasping, equilibrium and coordination were found also impaired by HI. Again, for the first time, we show the relevance of “sex and age” factorial interaction as well as the comparative degree of functional severity. Spontaneous functional recovery in motor coordination and righting, geotaxis and gait reflexes were found in some of those previous works (Lubics et al., 2005; Karalis et al., 2011). In our case, in spite of the high maturation of motor systems in adults, these tasks were more demanding for them, due to increased body size vs. the rods widths. This could explain that functional motor recovery was only seen for HI-induced hyperactivity in males. Thus, HI-males spontaneously returned to the normal values shown by sham animals, but deficient reflexes were still evident at 70 days of age.

On the other hand, this persistence of sensorimotor impairments until adulthood makes the beneficial effects induced by NH more remarkable, mostly in the reflex responses. This early life tactile and proprioceptive sensory stimulation reversed not only the impairment in reflexes but also exerted beneficial effects on coordination and equilibrium. Senses of touch, balance and proprioception are the first of the seven sensory systems developed during ontogeny. Thus, in the present work, the improvement of the vestibular system can be considered a notorious effect of NH, as compared to sensory outputs achieved in other studies (Paolucci et al., 2015). It is likely that the handling protocol, that involves the animals being held by the experimenter and the pups individually resting in the cage, may constitute a scenario where the vestibular system is challenged, trained and reinforced. In fact, early tactile and vestibular stimulations were postulated as crucial for motor behavior development (i.e., Labarba et al., 1974; Clark et al., 1977) with the maturation rate of inhibitory systems (Oakley and Plotkin, 1975) as a hypothesis to explain hyperexcitability stages, such as the pop-corn behavior shown by mice (Wahlsten, 1974) and as also observed here, in terms of emotional behaviors, which can be modulated by sensorimotor training (Caston and Lateurte, 1997). In agreement with the pyramid of learning postulated by Taylor and Trott, 1991 (as cited in Williams and Shellenberger, 1994), an improvement in the sensory integration dysfunction induced by HI, mostly in males, should also facilitate the improvement in learning capacities, as shown by our results. In the histological analysis, the injury score of the underlying neuroanatomical areas was reduced, but further experiments with detailed evaluation of these areas will provide clues about this sensory integration hypothesis.

Regarding the motor development, although some authors have described no impairment in the CYT (Sanches et al., 2013a), most of them reported that HI causes a preference to use the unimpaired forepaw (Grow et al., 2003; Chang et al., 2005; Jones et al., 2008; Kim et al., 2008; Lee et al., 2010; van Velthoven et al., 2010; van der Kooij et al., 2010; Pazos et al., 2012), as was also shown in the present work. In agreement with literature (Fan et al., 2011, 2013), no differences between male and female were observed. The poorer motor skills in children with neonatal encephalopathy compared to control could be related with the size of the CC (Van Kooij et al., 2008). This is important to note, since in the present work the CC is the area that shows, in both sexes, a statistical significant reduction of injury score in mice receiving NH.

The tertiary screening assessed locomotor activity and non-cognitive functions. It is well established that hippocampal areas are highly vulnerable to HI, and that hippocampal injury leads to hyperactivity (Shen et al., 1991). Most of the studies report that HI mice present hyperactivity in spontaneous ACTs or in the OF test (Balduini et al., 2000, 2001; Ten et al., 2004; McAuliffe et al., 2006; Arteni et al., 2010; Schlager et al., 2011; Rojas et al., 2013). Lubics et al. (2005) also detected that although HI were more active; when locomotion requires a higher level of coordination, mice can be hypoactive. Short test duration (Chou et al., 2001) and assessment in the light period (Antier et al., 1998) can also elicit reduced activity in HI rodents, probably because under these conditions they reflect an anxiogenic response. Thus, in the present work we show that the expression of neophobia and anxiety-like behaviors depend on the anxiogenic conditions of the test. In mild anxiogenic conditions the animals were found hyperactive, exhibiting a hyperexcitability stage, while with higher illumination they showed reduced exploratory activity. This was consistent with the behavioral responses shown in the other tests (the DLB and the performance in the long arm of the TM) and is also in agreement with other works (Girard et al., 2009, 2012; Carletti et al., 2012; Sanches et al., 2013a,b; Soares et al., 2013).

Academic performance and intellectual abilities are important aspects in children with neonatal encephalopathy (Robertson and Finer, 1988, 1993; Moster et al., 2002; van Handel et al., 2007). At a translational level, cognitive impairment has been reported many times, especially in the MWM. Learning impairments (Young et al., 1986; Ikeda et al., 2001; Ten et al., 2003; de Paula et al., 2009; Arteni et al., 2010) related to a longer time to escape in ischemic group (Balduini et al., 2001; Chou et al., 2001; Ten et al., 2004; Ikeda et al., 2006; Huang et al., 2009) and memory dysfunction in the probe trial (Ten et al., 2003, 2004; Huang et al., 2009). Like us, no impairments in swimming ability or speed have been observed in injured animals (Ikeda et al., 2001, 2006; Arteni et al., 2003). Arteni et al. (2010) described lateralized and sex-dependent behavioral and morphological effects of unilateral neonatal cerebral HI in the rat. In other works (Ikeda et al., 2001; Arteni et al., 2003) only animals that suffered a right HI injury performed worse in the working memory tasks. This lateralized effect could explain why, in our case, working memory is not affected in HI animals.

To the best of our knowledge, there are no reports regarding sex differences in the functional recovery following HI in neonatal handled animals. Nesting environment (Mason et al., 2018), rehabilitative training (Tsuji et al., 2010) or early-life interventions based on environmental rearing conditions (Pereira et al., 2007, 2008; Fan et al., 2011; Rojas et al., 2013, 2015; Nie et al., 2016; Schuch et al., 2016) that share mechanisms of action with NH (Fernández-Teruel et al., 2002), also show sex-specific neuroprotection patterns. In these works, the sex differences analyzed in the recovery of HI after environmental enrichment or rehabilitative training in rats, described partial recovery in working memory in adolescent rats (Pereira et al., 2008) and improved swimming time and length in females but not in males after rehabilitative training (Tsuji et al., 2010). Thus, it is remarkable to note that the above mentioned studies showed sex-specific neuroprotection patterns, but with female sex as the most resilient, while males seemed to be less responsive to the interventions. On the other hand, different protocols for maternal separation lead to distinct behavioral outputs, from behavioral protection without morphological changes (Chou et al., 2001) or reduction of hippocampal CA volume (Lehmann et al., 2002) to worsening of the effects of neonatal HI (Tata et al., 2015). In other experimental models of aging, neurological and psychiatric diseases, NH has also demonstrated positive effects in behavior, such as a reduction of anxiety-like behavior or an improvement in learning and memory (Levine and Otis, 1958; Alasmi et al., 1997; Gschanes et al., 1998; Raineki et al., 2014). Also, we have previously shown that NH has long-term effects on reducing the impact of N-Methyl-D-aspartate (NMDA) excitotoxicity, reducing the incidence of seizures, their number and severity in rats psychogenetically selected for high- and low-avoidance (Fernández-Teruel et al., 2002). Furthermore, we have proved that the behavioral outcome in brain damage related to Alzheimer’s disease can be modulated by NH, in both males and females at adulthood (Cañete et al., 2015) and even at very advanced stages of disease in 17-month-old triple-transgenic mice (Torres-Lista and Giménez-Llort, 2015).

Many studies reported morphological, biophysical and biochemical changes following HI brain insult, especially in ipsilateral cerebral cortex, hippocampus, striatum and thalamus, after arterial occlusion (Towfighi et al., 1991; Huang and Castillo, 2008 ; and our own precedent work Shrivastava et al., 2012), but there is scarcity of data to understand the consequential behavioral changes. Similarly, although morphological neuroprotective action in the hippocampus was reported after tactile stimulation (Rodrigues et al., 2004), no behavioral outcomes were evaluated. Therefore, in the present study, we also aimed to estimate the translation of the injury score on function for both risk (HI) and protection (NH) interventions. We looked for meaningful correlations related to behavioral variables, showing the functional impact of damage due to brain injury and its protection by NH. On the one hand, the analysis of neuropathological correlates shows that the level of damage induced/restored, measured in terms of atrophy, neuronal densities or cellularity in the affected areas, can be functionally correlated with behavioral variables. On the other hand, the behavioral correlates referred to changes in the five main behavioral domains of the pyramid of learning (i.e., physical/motor, sensory, behavioral, emotional and cognitive). The analysis identified the hippocampus as the most affected area, which could explain why it was difficult to completely reverse all the cognitive deficits in females. Caudate/putamen, thalamus and CC showed the highest percentages of prevention that may underline the better behavioral outcome in tasks dependent on these areas.

Concerning the translation of the experimental NH administered to mice from PND1, it could model an early tactile and proprioceptive sensory stimulation implemented on preterm infants. Since the functional sensory response of preterm children is immature (Fitzgerald, 2005), the clinical benefits may apply more specifically to older preterm infants (>30 weeks gestational age) or HI infants post hypothermia treatment. The neuroprotective effect of early-life stimulation could also be important during the pregnancy or prenatal period, as considered by Netto et al. (2018) and Durán-Carabali et al. (2017). At the clinical level, the standard of care in cases of moderate to severe HIE is therapeutic hypothermia which has been demonstrated to increase long-term survival without disability (Tagin et al., 2012). Despite the efficiency of hypothermia, it is not enough to prevent all injury or neurological symptoms. Brain damage in term newborn infants therefore remains a clinical problem due to there being limited therapeutic outcomes and since research is constrained by obvious ethical limitations. The therapeutical approaches investigating how to prevent or minimize the consequences of the HI insult have reported efficacy of handling depending on the severity of the damage (Chou et al., 2001). In our study, we demonstrate for the first time that, under similar injury conditions, males are the sex with better responsiveness to this early life intervention, mostly at the neuropathological level, as shown by the injury scores and the different number of areas protected. To a lesser extent, this protective effect on the neuropathological consequences of HI insult also has a functional impact on the behavioral output. This was shown by the better performances in some tasks and the neuropathological correlates that point out distinct neuronal substrates underlying the sex- and age- related functional impacts of these risk/protection interventions on sensorimotor, behavioral and cognitive outcomes from ontogeny to adulthood.

In conclusion, HI brain damage affected motor development and sensorimotor functions, and induced hyperactivity at weaning; anxiety-like behaviors and cognitive deficits during the adulthood in a sex- and age-selective manner. At the functional level, handling reversed the impaired reflex responses and allowed improvement in memory performances in the hippocampal-dependent spatial learning test (MWM), in males. At an individual level, remarkable neurological protection elicited by NH correlated with improved functional capacities. Strong correlations were found between the sensorimotor, behavioral and cognitive outcomes and the injury scores based on atrophy, neuronal densities and cellularity in the different affected areas (hippocampus, caudate/putamen, thalamus, neocortex and CC). These neuropathological correlates point at distinct neuronal substrates underlying the functional capacity to meet task-dependent performance demands and neuroanatomical targets for recovery. Overall, the present results provide evidence on a therapeutical potential of early life interventions based on tactile and proprioceptive sensory stimulation in the newborns with brain injury. It supports those in the literature who defend the benefits of perinatal rearing conditions as being important to be considered as adjuvant to the current treatments. Moreover, it shows a sex-specificity that benefits male sex, who were more at risk and reported to be less responsible to most interventions.

## Ethics Statement

All experimental procedures were approved by the Ethical Committee of Universitat Autònoma de Barcelona (CEEAH 811) in accordance with Spanish regulations and the European Communities Council Directives (2010/63/UE).

## Author Contributions

LG-L conceived and designed the experiments. KS and MR performed the risk/protection strategies. KS performed and analyzed the neuropathological studies. AM performed and analyzed the behavioral studies. LG-L, KS and AM wrote the manuscript. All authors revised and approved the final version of the manuscript. AM and KS equally contributed to the present work.

## Conflict of Interest Statement

The authors declare that the research was conducted in the absence of any commercial or financial relationships that could be construed as a potential conflict of interest.

## References

[B1] Alasmi M. M. Pickens W. L. Hoath S. B. (1997). Effect of tactile stimulation on serum lactate in the newborn rat. Pediatr. Res. 41, 857–861. 10.1203/00006450-199706000-00010 9167199

[B2] Andiné P. Thordstein M. Kjellmer I. Nordborg C. Thiringer K. Wennberg E.. (1990). Evaluation of brain damage in a rat model of neonatal hypoxic-ischemia. J. Neurosci. Methods 35, 253–260. 10.1016/0165-0270(90)90131-x 2084395

[B3] Antier D. Zhang B. L. Mailliet F. Akoka S. Pourcelot L. Sannajust F. (1998). Effects of neonatal focal cerebral hypoxia-ischemia on sleep-waking pattern, ECoG power spectra and locomotor activity in the adult rat. Brain Res. 807, 29–37. 10.1016/s0006-8993(98)00703-3 9756989

[B4] Arteni N. S. Pereira L. O. Rodrigues A. L. Lavinsky D. Achaval M. E. Netto C. A. (2010). Lateralized and sex-dependent behavioral and morphological effects of unilateral neonatal cerebral hypoxia-ischemia in the rat. Behav. Brain Res. 210, 92–98. 10.1016/j.bbr.2010.02.015 20156487

[B5] Arteni N. S. Salgueiro J. Torres I. Achaval M. Netto C. A. (2003). Neonatal cerebral hypoxia-ischemia causes lateralized memory impairments in the adult rat. Brain Res. 973, 171–178. 10.1016/s0006-8993(03)02436-3 12738060

[B6] Balduini W. De Angelis V. Mazzoni E. Cimino M. (2000). Long-lasting behavioral alterations following a hypoxic/ischemic brain injury in neonatal rats. Brain Res. 859, 318–325. 10.1016/s0006-8993(00)01997-1 10719080

[B7] Balduini W. De Angelis V. Mazzoni E. Cimino M. (2001). Simvastatin protects against long-lasting behavioral and morphological consequences of neonatal hypoxic/ischemic brain injury. Stroke 32, 2185–2191. 10.1161/hs0901.094287 11546915

[B8] Bona E. Hagberg H. Løberg E. M. Bågenholm R. Thoresen M. (1998). Protective effects of moderate hypothermia after neonatal Hypoxia-Ischemia: short- and long-term outcome. Pediatr. Res. 43, 738–745. 10.1203/00006450-199806000-00005 9621982

[B9] Bona E. Johansson B. B. Hagberg H. (1997). Sensorimotor function and neuropathology five to six weeks after hypoxia-ischemia in seven-day-old rats. Pediatr. Res. 42, 678–683. 10.1203/00006450-199711000-00021 9357943

[B10] Cañete T. Blázquez G. Tobeña A. Giménez-Llort L. Fernández-Teruel A. (2015). Cognitive and emotional alterations in young Alzheimer’s disease (3xTgAD) mice: effects of neonatal handling stimulation and sexual dimorphism. Behav. Brain Res. 281, 156–171. 10.1016/j.bbr.2014.11.004 25446741

[B11] Carletti J. V. Deniz B. F. Miguel P. M. Rojas J. J. Kolling J. Scherer E. B.. (2012). Folic acid prevents behavioral impairment and Na^+^, K^+^-ATPase inhibition caused by neonatal hypoxia-ischemia. Neurochem. Res. 37, 1624–1630. 10.1007/s11064-012-0757-6 22528830

[B12] Caston J. Lateurte J. (1997). Effects of a brief sensorimotor training on the development of behavioral inhibition in the mouse. Behav. Processes 39, 295–298. 10.1016/s0376-6357(96)00754-1 24897337

[B13] Chang Y. S. Mu D. Wendland M. Sheldon R. A. Vexler Z. S. McQuillen P. S.. (2005). Erythropoietin improves functional and histological outcome in neonatal stroke. Pediatr. Res. 58, 106–111. 10.1203/01.pdr.0000163616.89767.69 15879287

[B14] Chou I.-C. Trakht T. Signori C. Smith J. Felt B. T. Vazquez D. M.. (2001). Behavioral/environmental intervention improves learning after cerebral hypoxia-ischemia in rats. Stroke 32, 2192–2197. 10.1161/hs0901.095656 11546916

[B15] Clark D. L. Kreutzberg J. R. Chee F. K. (1977). Vestibular stimulation influence on motor development in infants. Science 196, 1228–1229. 10.1126/science.300899 300899

[B16] de Paula S. Vitola A. S. Greggio S. de Paula D. Mello P. B. Lubianca J. M.. (2009). Hemispheric brain injury and behavioral deficits induced by severe neonatal hypoxia-ischemia in rats are not attenuated by intravenous administration of human umbilical cord blood cells. Pediatr. Res. 65, 631–635. 10.1203/PDR.0b013e31819ed5c8 19430381

[B17] Donders J. Hoffman N. M. (2002). Gender differences in learning and memory after pediatric traumatic brain injury. Neuropsychology 16, 491–499. 10.1037//0894-4105.16.4.491 12382988

[B18] Durán-Carabali L. E. Arcego D. M. Odorcyk F. K. Reichert L. Cordeiro J. L. Sanches E. F.. (2017). Prenatal and early postnatal environmental enrichment reduce acute cell death and prevent neurodevelopment and memory impairments in rats submitted to neonatal hypoxia ischemia. Mol. Neurobiol. 55, 3627–3641. 10.1007/s12035-017-0604-5 28523564

[B19] Elsmén E. Hansen Pupp I. Hellström-Westas L. (2004). Preterm male infants need more initial respiratory and circulatory support than female infants. Acta Paediatr. 93, 529–533. 10.1080/08035250410024998 15188982

[B21] Fan X. Heijnen C. J. van der Kooij M. A. Groenendaal F. van Bel F. (2011). Beneficial effect of erythropoietin on sensorimotor function and white matter after hypoxia-ischemia in neonatal mice. Pediatr. Res. 69, 56–61. 10.1203/PDR.0b013e3181fcbef3 20856165

[B20] Fan L.-W. Lin S. Pang Y. Lei M. Zhang F. Rhodes P. G.. (2005). Hypoxia-ischemia induced neurological dysfunction and brain injury in the neonatal rat. Behav. Brain Res. 165, 80–90. 10.1016/j.bbr.2005.06.033 16140403

[B22] Fan X. van Bel F. van der Kooij M. A. Heijnen C. J. Groenendaal F. (2013). Hypothermia and erythropoietin for neuroprotection after neonatal brain damage. Pediatr. Res. 73, 18–23. 10.1038/pr.2012.139 23085819

[B23] Fernández-Teruel A. Escorihuela R. M. Driscoll P. Tobeña A. Bättig K. (1991). Infantile (handling) stimulation and behavior in young Roman high- and low-avoidance rats. Physiol. Behav. 50, 563–565. 10.1016/0031-9384(91)90546-z 1801010

[B24] Fernández-Teruel A. Giménez-Llort L. Escorihuela R. M. Gil L. Aguilar R. Steimer T.. (2002). Early-life handling stimulation and environmental enrichment: are some of their effects mediated by similar neural mechanisms? Pharmacol. Biochem. Behav. 73, 233–245. 10.1016/S0091-3057(02)00787-6 12076742

[B25] Fitzgerald M. (2005). The development of nociceptive circuits. Nat. Rev. Neurosci. 6, 507–520. 10.1038/nrn1701 15995722

[B26] Fung E. B. Samson-Fang L. Stallings V. A. Conaway M. Liptak G. Henderson R. C.. (2002). Feeding dysfunction is associated with poor growth and health status in children with cerebral palsy. J. Am. Diet. Assoc. 102, 361–373. 10.1016/s0002-8223(02)90084-2 11902369

[B27] Giménez-Llort L. Blázquez G. Cañete T. Johansson B. Oddo S. Tobeña A.. (2007). Modeling behavioral and neuronal symptoms of Alzheimer’s disease in mice: a role for intraneuronal amyloid. Neurosci. Biobehav. Rev. 31, 125–147. 10.1016/j.neubiorev.2006.07.007 17055579

[B28] Giménez-Llort L. Fernández-Teruel A. Escorihuela R. M. Fredholm B. B. Tobeña A. Pekny M.. (2002). Mice lacking the adenosine A1 receptor are anxious and aggressive, but are normal learners with reduced muscle strength and survival rate. Eur. J. Neurosci. 16, 547–550. 10.1046/j.1460-9568.2002.02122.x 12193199

[B29] Girard S. Kadhim H. Beaudet N. Sarret P. Sébire G. (2009). Developmental motor deficits induced by combined fetal exposure to lipopolysaccharide and early neonatal hypoxia/ischemia: a novel animal model for cerebral palsy in very premature infants. Neuroscience 158, 673–682. 10.1016/j.neuroscience.2008.10.032 19010395

[B30] Girard S. Sébire H. Brochu M.-E. Briota S. Sarret P. Sébire G. (2012). Postnatal administration of IL-1Ra exerts neuroprotective effects following perinatal inflammation and/or hypoxic-ischemic injuries. Am. J. Perinatol. 26, 1331–1339. 10.1016/j.bbi.2012.09.001 22982341 PMC5023428

[B31] Grow J. L. Liu Y. Q. Barks J. D. E. (2003). Can lateralizing sensorimotor deficits be identified after neonatal cerebral hypoxia-ischemia in rats? Dev. Neurosci. 25, 394–402. 10.1159/000075665 14966380

[B32] Gschanes A. Eggenreich U. Windisch M. Crailsheim K. (1998). Early postnatal stimulation influences passive avoidance behaviour of adult rats. Behav. Brain Res. 93, 91–98. 10.1016/s0166-4328(97)00143-5 9659991

[B33] Hagberg H. Peebles D. Mallard C. (2002). Models of white matter injury: comparison of infectious, hypoxic-ischemic, and excitotoxic insults. Ment. Retard. Dev. Disabil. Res. Rev. 8, 30–38. 10.1002/mrdd.10007 11921384

[B34] Hagberg H. Wilson M. A. Matsushita H. Zhu C. Lange M. Gustavsson M.. (2004). PARP-1 gene disruption in mice preferentially protects males from perinatal brain injury. J. Neurochem. 90, 1068–1075. 10.1111/j.1471-4159.2004.02547.x 15312162

[B35] Hill C. A. Fitch R. H. (2012). Sex differences in mechanisms and outcome of neonatal hypoxia-ischemia in rodent models: implications for sex-specific neuroprotection in clinical neonatal practice. Neurol. Res. Int. 2012:867531. 10.1155/2012/867531 22474588 PMC3306914

[B36] Huang B. Y. Castillo M. (2008). Hypoxic-ischemic brain injury: imaging findings from birth to adulthood. Radiographics 28, 417–439. 10.1148/rg.282075066 18349449

[B37] Huang Z. Liu J. Cheung P.-Y. Chen C. (2009). Long-term cognitive impairment and myelination deficiency in a rat model of perinatal hypoxic-ischemic brain injury. Brain Res. 1301, 100–109. 10.1016/j.brainres.2009.09.006 19747899

[B38] Hurn P. D. Vannucci S. J. Hagberg H. (2005). Adult or perinatal brain injury: does sex matter? Stroke 36, 193–195. 10.1161/01.STR.0000153064.41332.f6 15625289

[B39] Ikeda T. Mishima K. Aoo N. Harada K. Liu A. X. Egashira N.. (2006). Rehabilitative training tasks improve spatial learning impairment in the water maze following hypoxic-ischemic insult in neonatal rats. Pediatr. Res. 59, 61–65. 10.1203/01.pdr.0000190582.49589.14 16326986

[B40] Ikeda T. Mishima K. Yoshikawa T. Iwasaki K. Fujiwara M. Xia Y. X.. (2001). Selective and long-term learning impairment following neonatal hypoxic-ischemic brain insult in rats. Behav. Brain Res. 118, 17–25. 10.1016/s0166-4328(00)00287-4 11163630

[B41] Jansen E. M. Low W. C. (1996). Long-term effects of neonatal ischemic-hypoxic brain injury on sensorimotor and locomotor tasks in rats. Behav. Brain Res. 78, 189–194. 10.1016/0166-4328(95)00248-0 8864051

[B42] Jones N. M. Kardashyan L. Callaway J. K. Lee E. M. Beart P. M. (2008). Long-term functional and protective actions of preconditioning with hypoxia, cobalt chloride, and desferrioxamine against hypoxic-ischemic injury in neonatal rats. Pediatr. Res. 63, 620–624. 10.1203/PDR.0b013e31816d9117 18317402

[B43] Karalis F. Soubasi V. Georgiou T. Nakas C. T. Simeonidou C. Guiba-Tziampiri O.. (2011). Resveratrol ameliorates hypoxia/ischemia-induced behavioral deficits and brain injury in the neonatal rat brain. Brain Res. 1425, 98–110. 10.1016/j.brainres.2011.09.044 22018692

[B44] Kim S. S. Lee K.-H. Sung D. K. Shim J. W. Kim M. J. Jeon G. W.. (2008). Erythropoietin attenuates brain injury, subventricular zone expansion, and sensorimotor deficits in hypoxic-ischemic neonatal rats. J. Korean Med. Sci. 23, 484–491. 10.3346/jkms.2008.23.3.484 18583887 PMC2526527

[B45] Kurinczuk J. J. White-Koning M. Badawi N. (2010). Epidemiology of neonatal encephalopathy and hypoxic-ischaemic encephalopathy. Early Hum. Dev. 86, 329–338. 10.1016/j.earlhumdev.2010.05.010 20554402

[B46] Labarba R. C. Fernandez B. White J. L. Stewart A. (1974). The effects of neonatal tactile stimulation on adult emotional reactivity in BALB-c mice. Dev. Psychobiol. 7, 393–398. 10.1002/dev.420070503 4426466

[B47] Lang U. E. Lang F. Richter K. Vallon V. Lipp H.-P. Schnermann J.. (2003). Emotional instability but intact spatial cognition in adenosine receptor 1 knock out mice. Behav. Brain Res. 145, 179–188. 10.1016/s0166-4328(03)00108-6 14529816

[B48] Lee J. A. Kim B. I. L. Jo C. H. Choi C. W. Kim E.-K. Kim H.-S.. (2010). Mesenchymal stem-cell transplantation for hypoxic-ischemic brain injury in neonatal rat Model. Pediatr. Res. 67, 42–46. 10.1203/PDR.0b013e3181bf594b 19745781

[B49] Lehmann J. Pryce C. R. Jongen-Rêlo A. L. Stöhr T. Pothuizen H. H. Feldon J. (2002). Comparison of maternal separation and early handling in terms of their neurobehavioral effects in aged rats. Neurobiol. Aging 23, 457–466. 10.1016/s0197-4580(01)00320-7 11959408

[B50] Levine S. (1957). Infantile experience and resistance to physiological stress. Science 126:405. 10.1126/science.126.3270.405 13467220

[B51] Levine S. Broadhurst P. L. (1963). Genetic and ontogenetic determinants of adult behavior in the rat. J. Comp. Physiol. Psychol. 56, 423–428. 10.1037/h0040285 13930172

[B53] Levine S. Haltmeyer G. C. Karas G. G. Denenberg V. H. (1967). Physiological and behavioral effects of infantile stimulation. Physiol. Behav. 2, 55–59. 10.1016/0031-9384(67)90011-x

[B52] Levine S. Otis L. S. (1958). The effects of handling before and after weaning on the resistance of albino rats to later deprivation. Can. J. Psychol. 12, 103–108. 10.1037/h0083730 13546994

[B54] Lubics A. Regldi D. Tamás A. Kiss P. Szalai M. Szalontay L.. (2005). Neurological reflexes and early motor behavior in rats subjected to neonatal hypoxic-ischemic injury. Behav. Brain Res. 157, 157–165. 10.1016/j.bbr.2004.06.019 15617782

[B55] Mallard C. Vexler Z. S. (2015). Modeling ischemia in the immature brain: how translational are animal models? Stroke 46, 3006–3011. 10.1161/strokeaha.115.007776 26272384 PMC4589478

[B56] Månsson J. Fellman V. Stjernqvist K. EXPRESS Study Group (authors). (2015). Extremely preterm birth affects boys more and socio-economic and neonatal variables pose sex-specific risks. Acta Paediatr. 104, 514–521. 10.1111/apa.12937 25620552

[B57] Marlow N. Rose A. S. Rands C. E. Draper E. S. (2005). Neuropsychological and educational problems at school age associated with neonatal encephalopathy. Arch. Dis. Child. Fetal Neonatal Ed. 90, F380–F387. 10.1136/adc.2004.067520 16113154 PMC1721935

[B59] Mason B. Rollins L. G. Asumadu E. Cange C. Walton N. Donaldson S. T. (2018). Nesting environment provides sex-specific neuroprotection in a rat model of neonatal hypoxic-ischemic injury. Front. Behav. Neurosci. 12:221. 10.3389/fnbeh.2018.00221 30356904 PMC6190890

[B60] McAuliffe J. J. Miles L. Vorhees C. V. (2006). Adult neurological function following neonatal hypoxia-ischemia in a mouse model of the term neonate: water maze performance is dependent on separable cognitive and motor components. Brain Res. 1118, 208–221. 10.1016/j.brainres.2006.08.030 16997287

[B61] Millar L. J. Shi L. Hoerder-Suabedissen A. Molnár Z. (2017). Neonatal hypoxia ischaemia: mechanisms, models, and therapeutic challenges. Front. Cell. Neurosci. 11:78. 10.3389/fncel.2017.00078 28533743 PMC5420571

[B62] Montagu M. F. A. (1953). The sensory influences of the skin. Tex. Rep. Biol. Med. 11, 292—301. 13077397

[B63] Moster D. Lie R. T. Markestad T. (2002). Joint association of Apgar scores and early neonatal symptoms with minor disabilities at school age. Arch. Dis. Child. Fetal Neonatal Ed. 86, F16–F21. 10.1136/fn.86.1.f16 11815542 PMC1721350

[B64] Netto C. A. Sanches E. F. Odorcyk F. Duran-Carabali L. E. Sizonenko S. V. (2018). Pregnancy as a valuable period for preventing hypoxia-ischemia brain damage. Int. J. Dev. Neurosci. 70, 12–24. 10.1016/j.ijdevneu.2018.06.004 29920306

[B65] Netto C. A. Sanches E. Odorcyk F. K. Duran-Carabali L. E. Weis S. N. (2017). Sex-dependent consequences of neonatal brain hypoxia-ischemia in the rat. J. Neurosci. Res. 95, 409–421. 10.1002/jnr.23828 27870406

[B66] Nie X. Lowe D. W. Rollins L. G. Bentzley J. Fraser J. L. Martin R.. (2016). Sex-specific effects of N-acetylcysteine in neonatal rats treated with hypothermia after severe hypoxia-ischemia. Neurosci. Res. 108, 24–33. 10.1016/j.neures.2016.01.008 26851769 PMC4903952

[B67] Northington F. J. (2006). Brief update on animal models of hypoxic-ischemic encephalopathy and neonatal stroke. ILAR J. 47, 32–38. 10.1093/ilar.47.1.32 16391429

[B68] Oakley D. A. Plotkin H. C. (1975). Ontogeny of spontaneous locomotor activity in rabbit, rat, and guinea pig. J. Comp. Physiol. Psychol. 89, 267–273. 10.1037/h0076816 1150966

[B69] Paolucci T. Piccinini G. Paolucci S. Spadini E. Saraceni V. M. Morone G. (2015). Tactile and proprioceptive sensory stimulation modifies estimation of walking distance but not upright gait stability: a pilot study. J. Phys. Ther. Sci. 27, 3287–3293. 10.1589/jpts.27.3287 26644695 PMC4668186

[B70] Pazos M. R. Cinquina V. Gómez A. Layunta R. Santos M. Fernández-Ruiz J.. (2012). Cannabidiol administration after hypoxia-ischemia to newborn rats reduces long-term brain injury and restores neurobehavioral function. Neuropharmacology 63, 776–783. 10.1016/j.neuropharm.2012.05.034 22659086

[B71] Peacock J. L. Marston L. Marlow N. Calvert S. A. Greenough A. (2012). Neonatal and infant outcome in boys and girls born very prematurely. Pediatr. Res. 71, 305–310. 10.1038/pr.2011.50 22258087

[B72] Pereira L. O. Arteni N. S. Petersen R. C. da Rocha A. P. Achaval M. Netto C. A. (2007). Effects of daily environmental enrichment on memory deficits and brain injury following neonatal hypoxia-ischemia in the rat. Neurobiol. Learn. Mem. 87, 101–108. 10.1016/j.nlm.2006.07.003 16931063

[B73] Pereira L. O. Strapasson A. C. P. Nabinger P. M. Achaval M. Netto C. A. (2008). Early enriched housing results in partial recovery of memory deficits in female, but not in male, rats after neonatal hypoxia-ischemia. Brain Res. 1218, 257–266. 10.1016/j.brainres.2008.04.010 18514167

[B74] Platt M. J. Cans C. Johnson A. Surman G. Topp M. Torrioli M. G.. (2007). Trends in cerebral palsy among infants of very low birthweight (<1500 g) or born prematurely (<32 weeks) in 16 European centres: a database study. Lancet 369, 43–50. 10.1136/adc.2007.117689 17208641

[B75] Raineki C. Lucion A. B. Weinberg J. (2014). Neonatal handling: an overview of the positive and negative effects. Dev. Psychobiol. 56, 1613–1625. 10.1002/dev.21241 25132525 PMC4833452

[B76] Reilly S. Skuse D. Poblete X. (1996). Prevalence of feeding problems and oral motor dysfunction in children with cerebral palsy: a community survey. J. Pediatr. 129, 877–882. 10.1016/s0022-3476(96)70032-x 8969730

[B77] Rice J. E. III. Vannucci R. C. Brierley J. B. (1981). The influence of immaturity on hypoxic-ischemic brain damage in the rat. Ann. Neurol. 9, 131–141. 10.1002/ana.410090206 7235629

[B78] Robertson C. M. Finer N. N. (1993). Long-term follow-up of term neonates with perinatal asphyxia. Clin. Perinatol. 20, 483–500. 10.1016/s0095-5108(18)30405-6 7689432

[B79] Rodrigues A. L. Arteni N. S. Abel C. Zylbersztejn D. Chazan R. Viola G.. (2004). Tactile stimulation and maternal separation prevent hippocampal damage in rats submitted to neonatal hypoxia-ischemia. Brain Res. 1002, 94–99. 10.1016/j.brainres.2003.12.020 14988038

[B80] Rojas J. J. Deniz B. F. Miguel P. M. Diaz R. Hermel Edo E.-S. Achaval M.. (2013). Effects of daily environmental enrichment on behavior and dendritic spine density in hippocampus following neonatal hypoxia-ischemia in the rat. Exp. Neurol. 241, 25–33. 10.1016/j.expneurol.2012.11.026 23219882

[B81] Rojas J. J. Deniz B. F. Schuch C. P. Carletti J. V. Deckmann I. Diaz R.. (2015). Environmental stimulation improves performance in the ox-maze task and recovers Na^+^,K^+^-ATPase activity in the hippocampus of hypoxic-ischemic rats. Neuroscience 291, 118–127. 10.1016/j.neuroscience.2015.01.017 25617656

[B82] Rutter M. Caspi A. Moffitt T. E. (2003). Using sex differences in psychopathology to study causal mechanisms: unifying issues and research strategies. J. Child Psychol. Psychiatry 44, 1092–1115. 10.1111/1469-7610.00194 14626453

[B83] Sanches E. F. Arteni N. S. Nicola F. Boisserand L. Willborn S. Netto C. A. (2013a). Early hypoxia-ischemia causes hemisphere and sex-dependent cognitive impairment and histological damage. Neuroscience 237, 208–215. 10.1016/j.neuroscience.2013.01.066 23395861

[B84] Sanches E. F. Arteni N. S. Scherer E. B. Kolling J. Nicola F. Willborn S.. (2013b). Are the consequences of neonatal hypoxia-ischemia dependent on animals’ sex and brain lateralization? Brain Res. 1507, 105–114. 10.1016/j.brainres.2013.02.040 23466455

[B85] Sanches E. F. Arteni N. Nicola F. Aristimunha D. Netto C. A. (2015). Sexual dimorphism and brain lateralization impact behavioral and histological outcomes following hypoxia-ischemia in P3 and P7 rats. Neuroscience 290, 581–593. 10.1016/j.neuroscience.2014.12.074 25620049

[B86] Schallert T. Fleming S. M. Leasure J. L. Tillerson J. L. Bland S. T. (2000). CNS plasticity and assessment of forelimb sensorimotor outcome in unilateral rat models of stroke, cortical ablation, parkinsonism and spinal cord injury. Neuropharmacology 39, 777–787. 10.1016/s0028-3908(00)00005-8 10699444

[B87] Schlager G. W. Griesmaier E. Wegleiter K. Neubauer V. Urbanek M. Kiechl-Kohlendorfer U.. (2011). Systemic G-CSF treatment does not improve long-term outcomes after neonatal hypoxic-ischaemic brain injury. Exp. Neurol. 230, 67–74. 10.1016/j.expneurol.2010.11.021 21145889

[B88] Schuch C. P. Diaz R. Deckmann I. Rojas J. J. Deniz B. F. Pereira L. O. (2016). Early environmental enrichment affects neurobehavioral development and prevents brain damage in rats submitted to neonatal hypoxia-ischemia. Neurosci. Lett. 617, 101–107. 10.1016/j.neulet.2016.02.015 26872850

[B89] Semple B. D. Blomgren K. Gimlin K. Ferriero D. M. Noble-Haeusslein L. J. (2013). Brain development in rodents and humans: identifying benchmarks of maturation and vulnerability to injury across species. Prog. Neurobiol. 106–107, 1–16. 10.1016/j.pneurobio.2013.04.001 23583307 PMC3737272

[B90] Sheldon R. A. Hall J. J. Noble L. J. Ferriero D. M. (2001). Delayed cell death in neonatal mouse hippocampus from hypoxia-ischemia is neither apoptotic nor necrotic. Neurosci. Lett. 304, 165–168. 10.1016/s0304-3940(01)01788-8 11343828

[B91] Sheldon R. A. Sedik C. Ferriero D. M. (1998). Strain-related brain injury in neonatal mice subjected to hypoxia-ischemia. Brain Res. 810, 114–122. 10.1016/s0006-8993(98)00892-0 9813271

[B92] Shen Y. Isaacson R. L. Smotherman W. P. (1991). The behavioral and anatomical effects of prenatal umbilical cord clamping in the rat and their alteration by the prior maternal administration of nimodipine. Restor. Neurol. Neurosci. 3, 11–22. 10.3233/RNN-1991-3102 21551629

[B93] Shrivastava K. Chertoff M. Llovera G. Recasens M. Acarin L. (2012). Short and long-term analysis and comparison of neurodegeneration and inflammatory cell response in the ipsilateral and contralateral hemisphere of the neonatal mouse brain after hypoxia/ischemia. Neurol. Res. Int. 2012:781512. 10.1155/2012/781512 22701792 PMC3372286

[B94] Smith A. L. Alexander M. Rosenkrantz T. S. Sadek M. L. Fitch R. H. (2014). Sex differences in behavioral outcome following neonatal hypoxia ischemia: insights from a clinical meta-analysis and a rodent model of induced hypoxic ischemic brain injury. Exp. Neurol. 254, 54–67. 10.1016/j.expneurol.2014.01.003 24434477

[B95] Soares L. M. Schiavon A. P. Milani H. de Oliveira R. M. W. (2013). Cognitive impairment and persistent anxiety-related responses following bilateral common carotid artery occlusion in mice. Behav. Brain Res. 249, 28–37. 10.1016/j.bbr.2013.04.010 23602921

[B96] Spandou E. Papadopoulou Z. Soubasi V. Karkavelas G. Simeonidou C. Pazaiti A.. (2005). Erythropoietin prevents long-term sensorimotor deficits and brain injury following neonatal hypoxia-ischemia in rats. Brain Res. 1045, 22–30. 10.1016/j.brainres.2005.03.013 15910759

[B97] Sullivan P. B. Lambert B. Rose M. Ford-Adams M. Johnson A. Griffiths P. (2000). Prevalence and severity of feeding and nutritional problems in children with neurological impairment: oxford feeding study. Dev. Med. Child Neurol. 42, 674–680. 10.1111/j.1469-8749.2000.tb00678.x 11085295

[B98] Tagin M. A. Woolcott C. G. Vincer M. J. Whyte R. K. Stinson D. A. (2012). Hypothermia for neonatal hypoxic ischemic encephalopathy. Arch. Pediatr. Adolesc. Med. 166, 558–566. 10.1001/archpediatrics.2011.1772 22312166

[B99] Tata D. A. Markostamou I. Ioannidis A. Gkioka M. Simeonidou C. Anogianakis G.. (2015). Effects of maternal separation on behavior and brain damage in adult rats exposed to neonatal hypoxia-ischemia. Behav. Brain Res. 280, 51–61. 10.1016/j.bbr.2014.11.033 25433094

[B100] Ten V. S. Bradley-Moore M. Gingrich J. A. Stark R. I. Pinsky D. J. (2003). Brain injury and neurofunctional deficit in neonatal mice with hypoxic-ischemic encephalopathy. Behav. Brain Res. 145, 209–219. 10.1016/s0166-4328(03)00146-3 14529818

[B101] Ten V. S. Wu E. X. Tang H. Bradley-Moore M. Fedarau M. V. Ratner V. I.. (2004). Late measures of brain injury after neonatal hypoxia-ischemia in mice. Stroke 35, 2183–2188. 10.1161/01.str.0000137768.25203.df 15272130

[B102] Thorngren-Jerneck K. Ohlsson T. G. Sandell A. Erlandsson K. Strand S.-E. Ryding E.. (2001). Cerebral glucose metabolism measured by positron emission tomography in term newborn infants with hypoxic ischemic encephalopathy. Pediatr. Res. 49, 495–501. 10.1203/00006450-200104000-00010 11264432

[B103] Tioseco J. A. Aly H. Essers J. Patel K. El-Mohandes A. A. E. (2006). Male sex and intraventricular hemorrhage. Pediatr. Crit. Care Med. 7, 40–44. 10.1097/01.pcc.0000192341.67078.61 16395073

[B104] Torres-Lista V. Giménez-Llort L. (2015). Early postnatal handling and environmental enrichment improve the behavioral responses of 17-month-old 3xTg-AD and non-transgenic mice in the Forced Swim Test in a gender-dependent manner. Behav. Processes 120, 120–127. 10.1016/j.beproc.2015.09.011 26431900

[B105] Towfighi J. Yager J. Y. Housman C. Vannucci R. C. (1991). Neuropathology of remote hypoxic-ischemic damage in the immature rat. Acta Neuropathol. 81, 578–587. 10.1007/bf00310141 1858486

[B106] Tsuji M. Aoo N. Harada K. Sakamoto Y. Akitake Y. Irie K.. (2010). Sex differences in the benefits of rehabilitative training during adolescence following neonatal hypoxia-ischemia in rats. Exp. Neurol. 226, 285–292. 10.1016/j.expneurol.2010.09.002 20833167

[B107] van der Kooij M. A. Ohl F. Arndt S. S. Kavelaars A. van Bel F. Heijnen C. J. (2010). Mild neonatal hypoxia-ischemia induces long-term motor- and cognitive impairments in mice. Brain Behav. Immun. 24, 850–856. 10.1016/j.bbi.2009.09.003 19748566

[B108] van Handel M. Swaab H. de Vries L. S. Jongmans M. J. (2007). Long-term cognitive and behavioral consequences of neonatal encephalopathy following perinatal asphyxia: a review. Eur. J. Pediatr. 166, 645–654. 10.1007/s00431-007-0437-8 17426984 PMC1914268

[B109] Van Kooij B. J. Van Handel M. Uiterwaal C. S. Groenendaal F. Nievelstein R. A. Rademaker K. J.. (2008). Corpus callosum size in relation to motor performance in 9- to 10-year-old children with neonatal encephalopathy. Pediatr. Res. 63, 103–108. 10.1203/pdr.0b013e31815b4435 18043516

[B110] van Velthoven C. T. J. Kavelaars A. van Bel F. Heijnen C. J. (2010). Nasal administration of stem cells: a promising novel route to treat neonatal ischemic brain damage. Pediatr. Res. 68, 419–422. 10.1203/pdr.0b013e3181f1c289 20639794

[B113] Vannucci S. J. Hagberg H. (2004). Hypoxia-ischemia in the immature brain. J. Exp. Biol. 207, 3149–3154. 10.1242/jeb.01064 15299036

[B111] Vannucci R. C. Vannucci S. J. (1997). A model of perinatal hypoxic-ischemic brain damage. Ann. N Y Acad. Sci. 835, 234–249. 10.1111/j.1749-6632.1997.tb48634.x 9616778

[B112] Vannucci R. C. Vannucci S. J. (2005). Perinatal hypoxic-ischemic brain damage: evolution of an animal model. Dev. Neurosci. 27, 81–86. 10.1159/000085978 16046840

[B114] Wahlsten D. (1974). A developmental time scale for postnatal changes in brain and behaviour of B6D2F_2 _ mice. Brain Res. 72, 251–264. 10.1016/0006-8993(74)90863-4 4838375

[B115] Williams M. S. Shellenberger S. (1994). How Does Your Engine Run? Albuquerque, NM: Therapy Works.

[B116] Young R. S. Kolonich J. Woods C. L. Yagel S. K. (1986). Behavioral performance of rats following neonatal hypoxia-ischemia. Stroke 17, 1313–1316. 10.1161/01.str.17.6.1313 3810735

[B117] Robertson C. M. Finer N. N. (1988). Educational readiness of survivors of neonatal encephalopathy associated with birth asphyxia at term. J. Dev. Behav. Pediatr. 9, 298–306. 10.1097/00004703-198810000-00009 2976068

